# Synthesis of Conjugated Tris- and Tetrakis (Carbazolyl) Azulenes with Intense Emission in the Visible Range

**DOI:** 10.3390/molecules30132797

**Published:** 2025-06-28

**Authors:** Amantay Iskanderov, Nurlan Merkhatuly, Ablaykhan Iskanderov, Saltanat Abeuova, Pavel Vojtisek

**Affiliations:** 1 Laboratory of Organic Semiconductor Chemistry, Karaganda Buketov University, Karaganda 100028, Kazakhstan; org.semicond@yandex.kz (A.I.); aby93@yandex.kz (A.I.); 2 The Higher School of Natural Sciences, Astana International University, Astana 020000, Kazakhstan; abeuova.salta@gmail.com; 3 Department of Inorganic Chemistry, Charles University, CZ-12800 Praha, Czech Republic

**Keywords:** azulene compounds, substituted azulenes, visible absorbances, fluorescent emission, substituted carbazoles, coupling reactions

## Abstract

New conjugated carbazolyl-substituted azulenes, such as 1,2,3-tris(carbazolyl)azulene and 1,2,3,6-tetrakis(carbazolyl)azulene, were synthesized via cross-coupling reactions in high yields. The resulting compounds exhibit a significant ability to absorb and emit light in the visible region, in the range of 400 to 600 nanometers. Studies have shown that azulene with carbazolyl substituents at positions 1, 2, 3, and 6 possesses unique photophysical properties, manifested as intense emission in the blue photoluminescence region (λ_PL_ at 444 and 490 nm), which is not observed in the original azulene. This feature arises due to the donor properties of carbazolyl substituents, which have a strong effect on the electronic structure of azulene, creating the conditions for a permitted HOMO-LUMO electronic transition.

## 1. Introduction

The widespread application of aromatic compounds possessing elongated π-electronic systems is due to their crucial role as functional materials in the field of organic optoelectronics.

In recent years, increasing attention has been focused on aromatic and heteroaromatic molecules, especially those containing aryl groups or substituents with electron-donor properties. For example, phenylamine naphthalenes and phenylamine anthracenes, due to their pronounced electron donor properties, attract considerable attention from scientists, since they have great potential for use in various fields of organic electronics [1,2,3,4].

Their application is considered in such promising areas as the development of organic semiconductors, catalysts for redox reactions, metal-organic frameworks (MOFs), and organic emitting diodes (OLEDs) [5,6,7,8,9,10].

One of the main advantages of these aromatic systems is their ability to precisely control the electronic structure of materials, which in turn leads to improved performance and optimized morphology.

In this regard, it is necessary to emphasize the importance of the structural isomer of naphthalene–azulene [11,12,13,14,15,16,17,18,19,20]. Its peculiarity lies in its unique aromatic structure, which determines its unique electronic and optical characteristics. Among them, a polarized structure with a dipole moment of 1.08 D [21] and anomalous fluorescence is particularly noteworthy. This fluorescence deviates from traditional concepts based on Kasha’s rule and manifests in a transition between the S_2_ and S_0_ energy levels [22,23,24].

**Figure 1:**
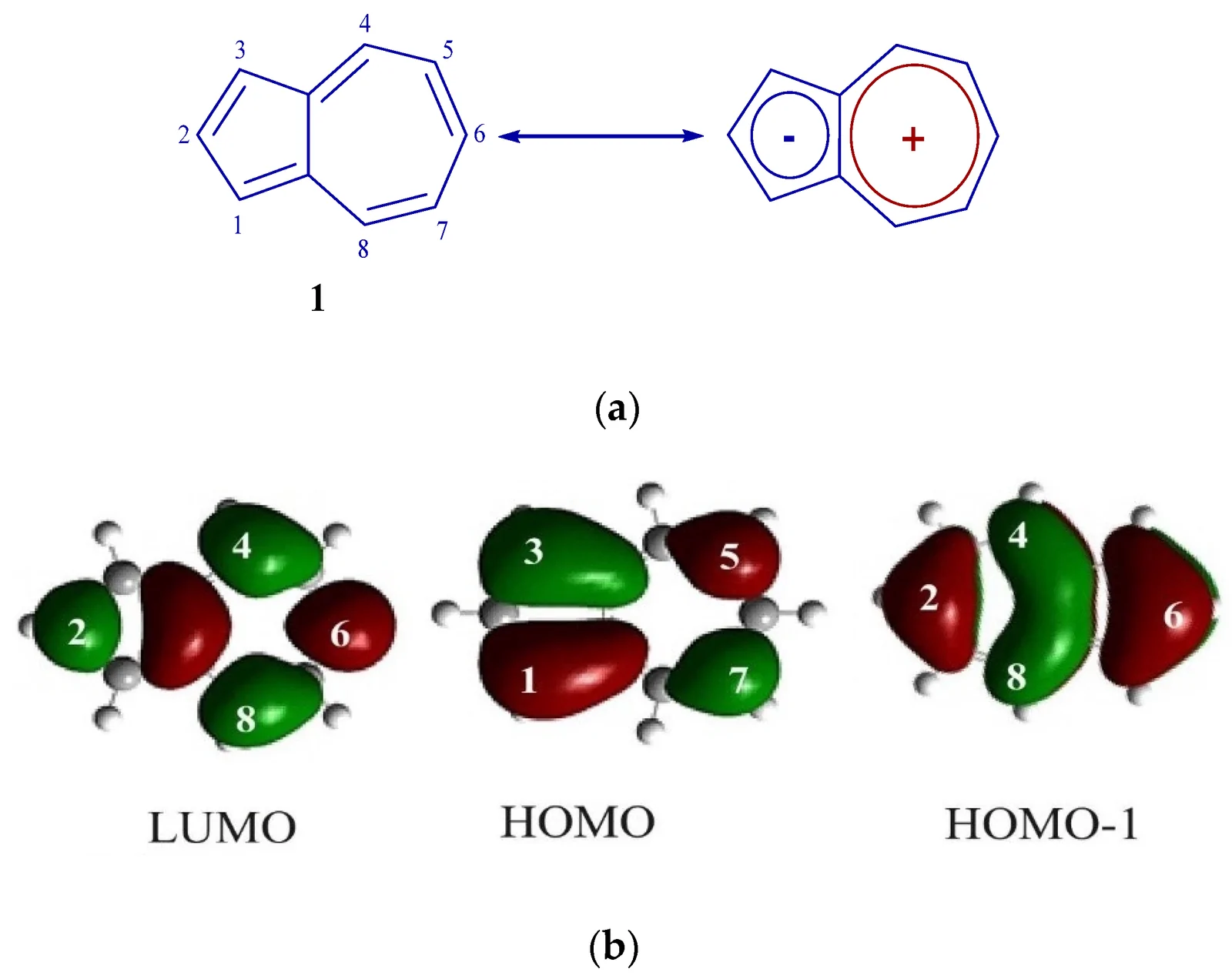
(**a**) Structure **1.** (**b**) FMO **1**.

Azulene **1** can be represented as a molecule formed by the condensation of the tropylium cation and the cyclopentadiene anion, which is clearly demonstrated in Figure 1a. It is precisely this unique molecular architecture that causes to the deviation of the HOMO and LUMO energy levels from the characteristics typical of standard aromatic hydrocarbons. In the azulene molecule, atoms located at positions 1 and 3, possess large HOMO coefficients, and carbon atoms C_2_ and C_6_ have large HOMO-1/LUMO (see Figure 1b).

A distinctive feature of azulene **1** compared to naphthalene is its saturated blue color, which indicates the absorption of light corresponding to the transition S_0_ → S_1_ (λ max is in the long-wave region of the spectrum at 580 nanometers). However, the molar absorption coefficient has a small value (350 M^−1^ cm^−1^), which indicates that it does not reach the level expected for the permitted π–π* transition [25].

Thus, modification of the structure of azulene by introducing functional groups, especially electron donor groups, can significantly change its electronic properties. This opens up ample opportunities for creating unique materials with unusual optical and electronic characteristics.

In this work, we demonstrate the synthesis of new carbazolyl-substituted azulenes. An effective cross-coupling method yielded 1,2,3-tris(carbazolyl)azulene **6** and 1,2,3,6-tetrakis(carbazolyl)azulene **10** in high yields. The study of photophysical properties of these molecular structures has shown that they lead to an allowed HOMO → LUMO electronic transition. This causes the appearance of intense absorption and luminescence spectra in the visible range.

## 2. Results and Discussions

**Scheme 1:**
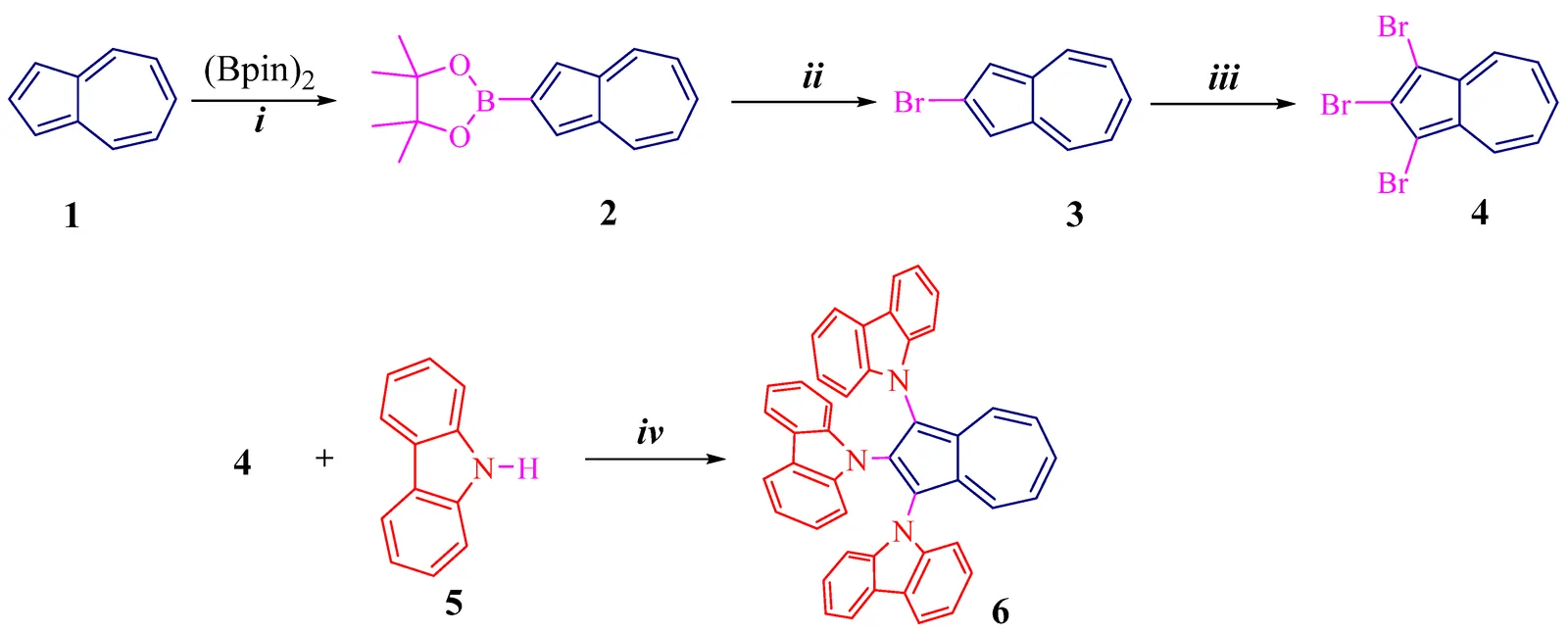
Preparation of **6**: (i) [Ir(cod)Cl]_2_, 2,2′-bpy, cyclohexane, reflux, 70%; (ii) CuBr, DMF, 90 °C, 72%; (iii) NBS, CH_2_Cl_2_, −20 °C, 92%; (iv) Pd(OAc)_2_, *t*-BuOK, *t*-Bu_3_PHBF_4_, toluene, reflux, 78%.

**Scheme 2:**
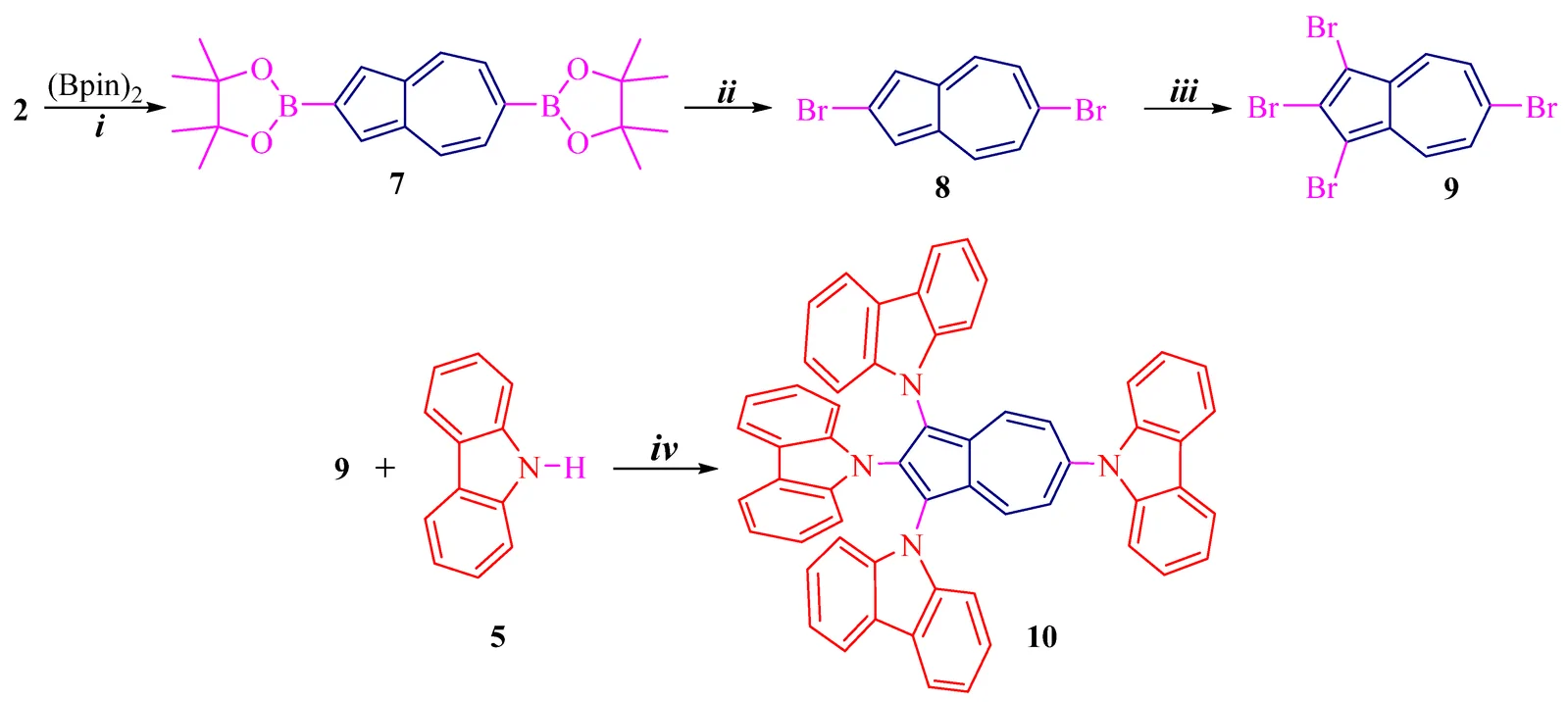
Preparation of **10**: (i) [Ir(cod)Cl]_2_, 2,2′-bpy, cyclohexane, reflux, 50%; (ii) CuBr, DMF, 90 °C, 70%; (iii) NBS, CH_2_Cl_2_, −20 °C, 90%; (iv) Pd(OAc)_2_, *t*-BuOK, *t*-Bu_3_PHBF_4_, toluene, reflux, 75%.

Scheme 1 and Scheme 2 illustrate synthetic routes for azulene conjugated compounds: 1,2,3-tri(carbazolyl)azulene **6** and 1,2,3,6-tetra(carbazolyl)azulene **10.**

As shown (Scheme 1), key molecule **2** was synthesized by direct borylation at the C_2_ position of **1** in the presence of an Ir-catalyst, according to the methodology described in the literature [26]. This compound is then successfully reacted with copper bromide to give bromoazulene **3** in 72% yield [27].

Next, the bromide **3** reacts with NBS (N-bromosuccinimide) to form tribromoazulene **4** in a high yield of 92%. The reaction proceeds regioselectively at the C_1_ and C_3_ positions.

Finally, the cross-coupling of tribromoazulene **4** and carbazole **5** in toluene, in the presence of a palladium catalyst, gives the final product, tris(carbazolyl)azulene **6**, in a high yield of 78%.

Another key molecule, **7**, was synthesized by selective C_6_ borylation of **2** under the above conditions, according to [28] (Scheme 2). Further reaction of diborylazulene **7** with copper bromide gives 2,6-dibromoazulene **8** in 70% yield [27,29]. The resulting dibromide **8** is then also reacted regioselectively at positions 1 and 3 with NBS to give tetrabromoazulene **9** in high yield of 90%. In a similar way, by cross-coupling reaction of tetrabromide **9** and **5**, the final product, tetrakis(carbazolyl)azulene **10**, was obtained. The yield is high, amounting to 75%.

The obtained tris(carbazolyl)azulene **6** and tetrakis(carbazolyl)azulene **10** are colored brown in contrast to the original **1**, which has a blue tint. These compounds are stable solids (Supplementary Materials, Section 3, Figure S13) that are easily soluble in organic solvents (CH_2_Cl_2_, CHCl_3_, C_6_H_5_CH_3,_ and C_6_H_5_Cl) at ambient temperature.

The structure and purity of synthesized compounds **6** and **10** were proved by NMR (^1^H and ^13^C), IR, and Mass spectrometry (Supplementary Materials).

**Figure 2:**
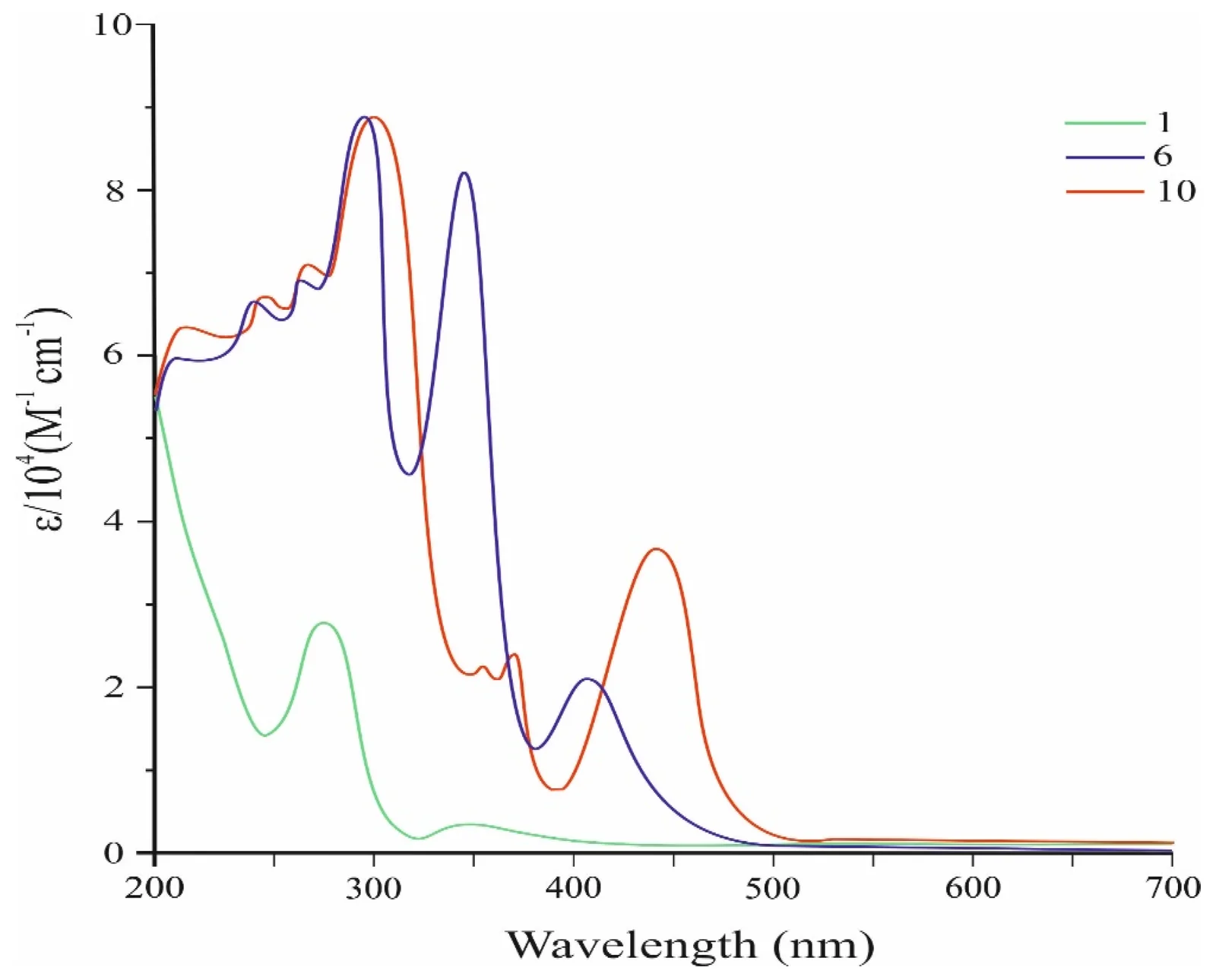
UV–Vis spectra of compounds **6**, **10**, and the original **1**.

Figure 2 shows the UV-Vis spectra of conjugated tris- and tetrakis(carbazolyl)azulenes **6** and **10**, while Table 1 presents their characteristics.

The absorption spectra of these compounds were analyzed against the spectrum of the initial molecule **1** (Figure 2).

As shown in Figure 2, the absorption spectrum of the compound **6** in dichloromethane solution shows the appearance of a new visible band with λmax at 407 nm (ɛ = 21.132 M^−1^ cm^−1^). The compound **10** also exhibits a strong visible absorption corresponding to the permitted π–π* electron transition with a maximum at 441 nm (ɛ = 36.666 M^−1^ cm^−1^).

The effective absorption of visible light in the compounds **6** and **10** is due to the strong electron donor effect of the carbazolyl groups. It should be noted that visible absorbances of **6** and **10** are significantly stronger than that of the original azulene **1**, which has a molar absorption coefficient in the visible region of only 350 M^−1^ cm^−1^ [25].

It is also seen from the spectrum that the visible absorption maximum of **10** is significantly (by 34 nm) shifted to the long-wave region with an increase in intensity compared to the absorption maximum of **6**. These changes may be due to a significant increase in π-conjugation and de-localization of electrons, which leads to narrowing of the HOMO–LUMO gap of molecule **10** (Figure 5).

**Figure 3:**
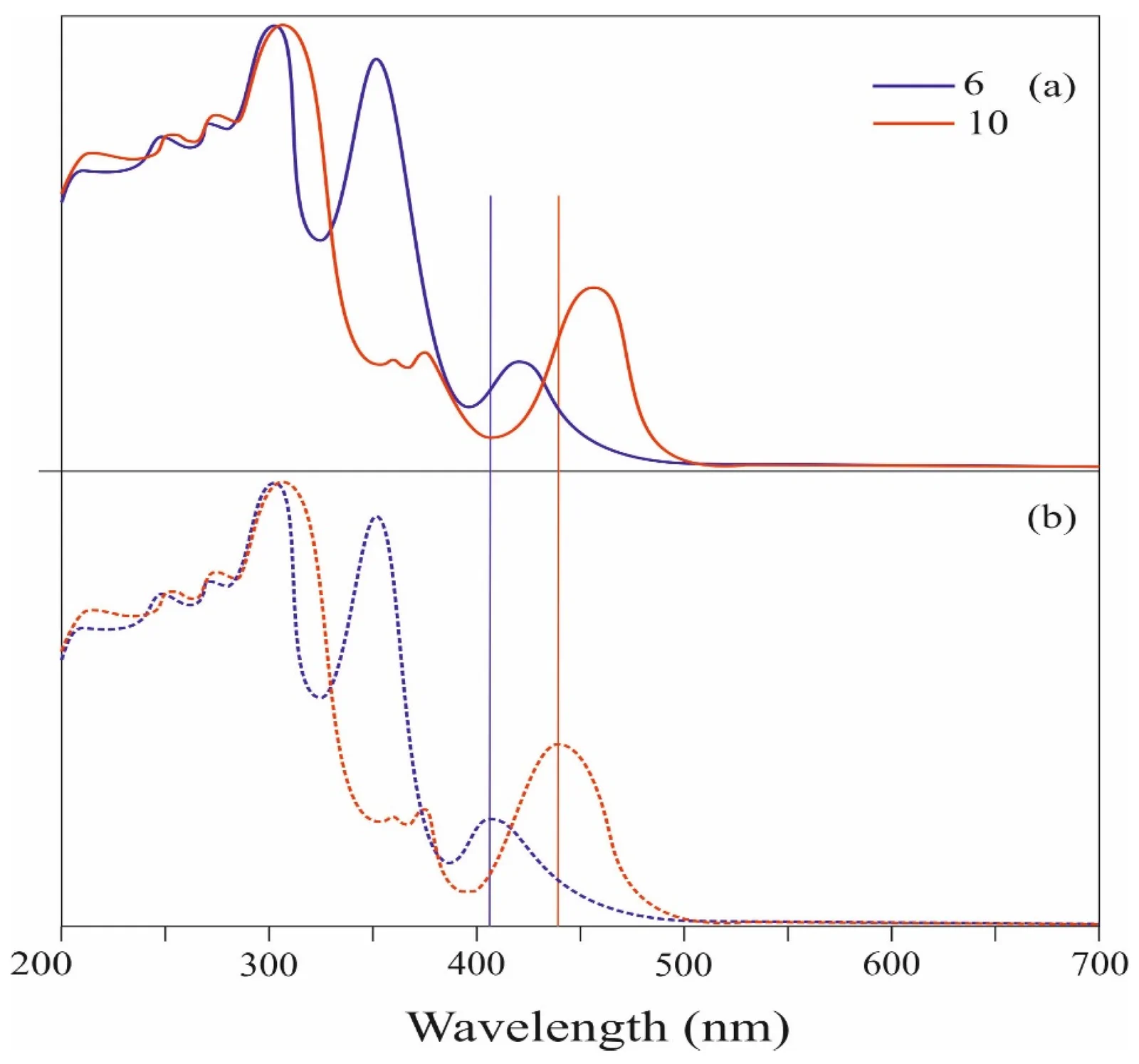
UV–Vis absorption spectra of **6** and **10**: (**a**) in film; (**b**) in CH_2_Cl_2_.

The study of the electronic absorbances of the compounds **6** and **10** was also carried out in the form of thin films (Supplementary Materials). Analysis of the spectra presented in Figure 3 shows that the absorption pattern of the compounds **6** and **10** in thin films is similar to their absorption in solution (see Figure 3b or Figure 2). However, the visible absorption maxima (λmax at 407 and 441 nm, respectively) for the compounds **6** and **10** are shifted to the long wavelength region by about 13 and 15 nanometers, respectively, compared to the solution. Redshift of spectral bands may be due to intermolecular interaction of compounds in the solid state.

It is worth noting that the absorption bands of compounds **6** and **10** observed in the spectrum coincide with the wavelength range (380–560 nm) characteristic of commercially available functional materials used in organic solar cells and organic dyes [30,31,32,33,34,35,36].

**Figure 4:**
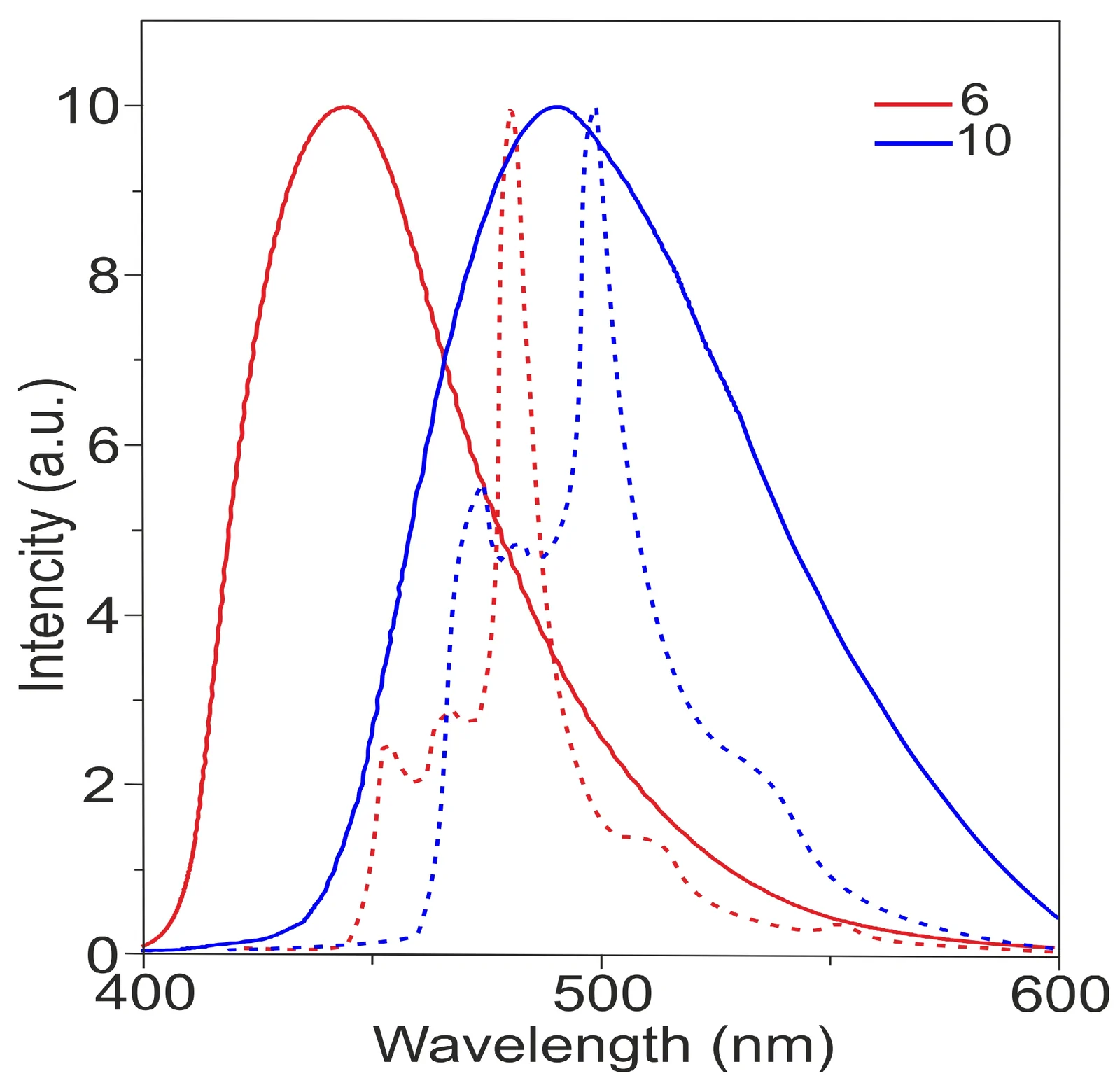
Photoluminescence (PL) spectra of compounds **6** and **10** in dichloromethane. Fluorescence spectra–solid lines, phosphorescence spectra–dashed lines.

Figure 4 shows the photoluminescence (PL) spectra of tris- and tetrakis (carbazolyl)azulenes **6** and **10** studied in solution, and Table 1 shows their characteristics.

The spectrum shows that compounds **6** and **10** exhibit new photoluminescence bands in the visible region with maxima at 411 and 437 nm. The quantum yields (Φ_PL_) are 12% and 25%, respectively (Table 1).

The fluorescence spectra of compounds **6** and **10** at room temperature are broad and structureless, while the phosphorescence spectra measured at 77 K show vibrational structures.

The analysis of Table 1 demonstrates that the most donor tetrasubstituted azulene **10** possesses lower S_1_ (2.87 eV) and T_1_ (2.63 eV) energies compared to the trisubstituted compound **6**. Thus, the S_1_ of compound **10** is significantly shifted towards lower energies by 0.19 eV. The T_1_ energy also has a shift to lower energies by 0.16 eV. As a result, the ΔE_ST_ of molecule **10** reaches 0.24 eV.

It is also observed that the lifetime (τ_p_/τ_d_) and the quantum yield of the fluorescence of compound **10** are superior to those of molecule **6** (Table 1).

So, the synthesized carbazolylazulenes **6** and **10** have a significant feature–intense photoluminescence in the visible part of the spectrum, which is not observed in the original azulene **1** [24,25].

Thus, studies of photophysical properties have shown that the introduction of electron-donor carbazolyl groups at positions 1,2,3, and 6 of azulene leads to the appearance of intense electronic absorptions (ε 21.132 M^−1^ cm^−1^ and ε 36.666 M^−1^ cm^−1^) and unique photoluminescent emissions (Φ_PL_ 12% and 25%) in the visible part of the spectrum.

**Figure 5:**
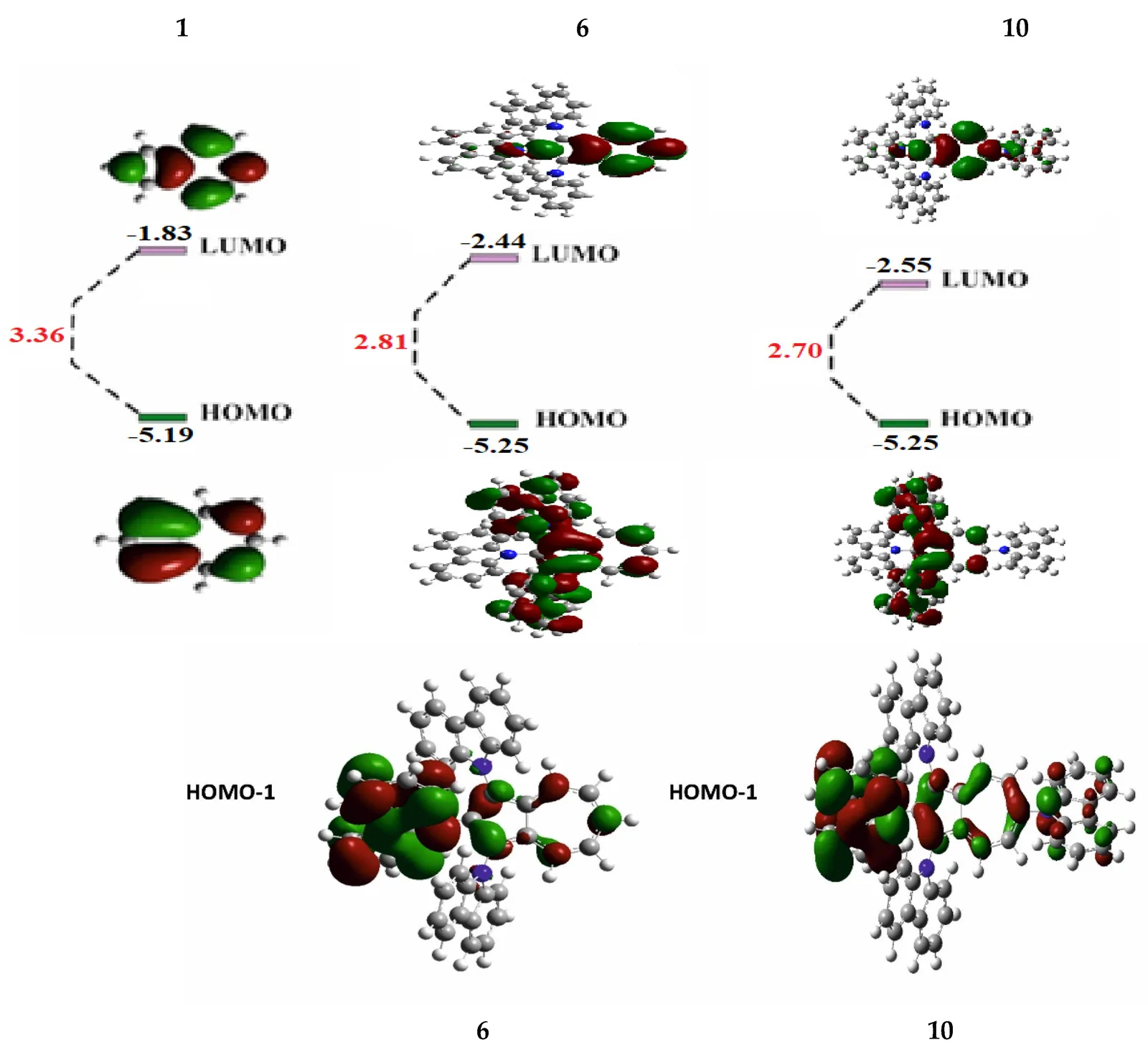
Distribution of frontal MO **6** and **10** versus azulene **1**.

To understand the electronic properties of the synthesized tris- and tetrakis(carbazolyl)azulenes **6** and **10** and the relationship between their structural configuration and optical behavior, DFT calculations (method B3LYP/6-31G*) were performed (Figure 5, Supplementary Materials).

Figure 5 shows that HOMO (and HOMO-1) of the compounds **6** and **10** are distributed throughout both the azulene structure and at the carbazolyl substituents.

This distribution of orbitals can occur as a result of interaction of HOMO-1 of azulene **1** with HOMO of carbazole **5** [29], because in HOMO of the original azulene C_2_ and C_6_ are nodal, while in HOMO-1 these atoms are not included in the nodal plane and have large atomic-orbital coefficients (Figure 1b).

Studies also revealed that HOMO (5.25 eV each) and LUMO (−2.44 eV and −2.55 eV) levels of the compounds **6** and **10** occupy lower energy positions compared to similar levels of the original azulene **1.**

At the same time, LUMO levels decrease significantly by 0.61 and 0.72 eV, respectively. As a result, the energy gaps between the frontal orbitals of these compounds narrow by 0.55 eV and 0.66 eV.

Probably, the decrease in FMO causes the permitted π–π* electronic transition in molecules **6** and **10**, in contrast to azulene **1**, where this transition is prohibited [25]. As a result, intense absorption and emission are observed in the visible spectrum (Figure 2 and Figure 4, data in Table 1), which indicates significant differences in the electronic structure of these compounds.

**Figure 6:**
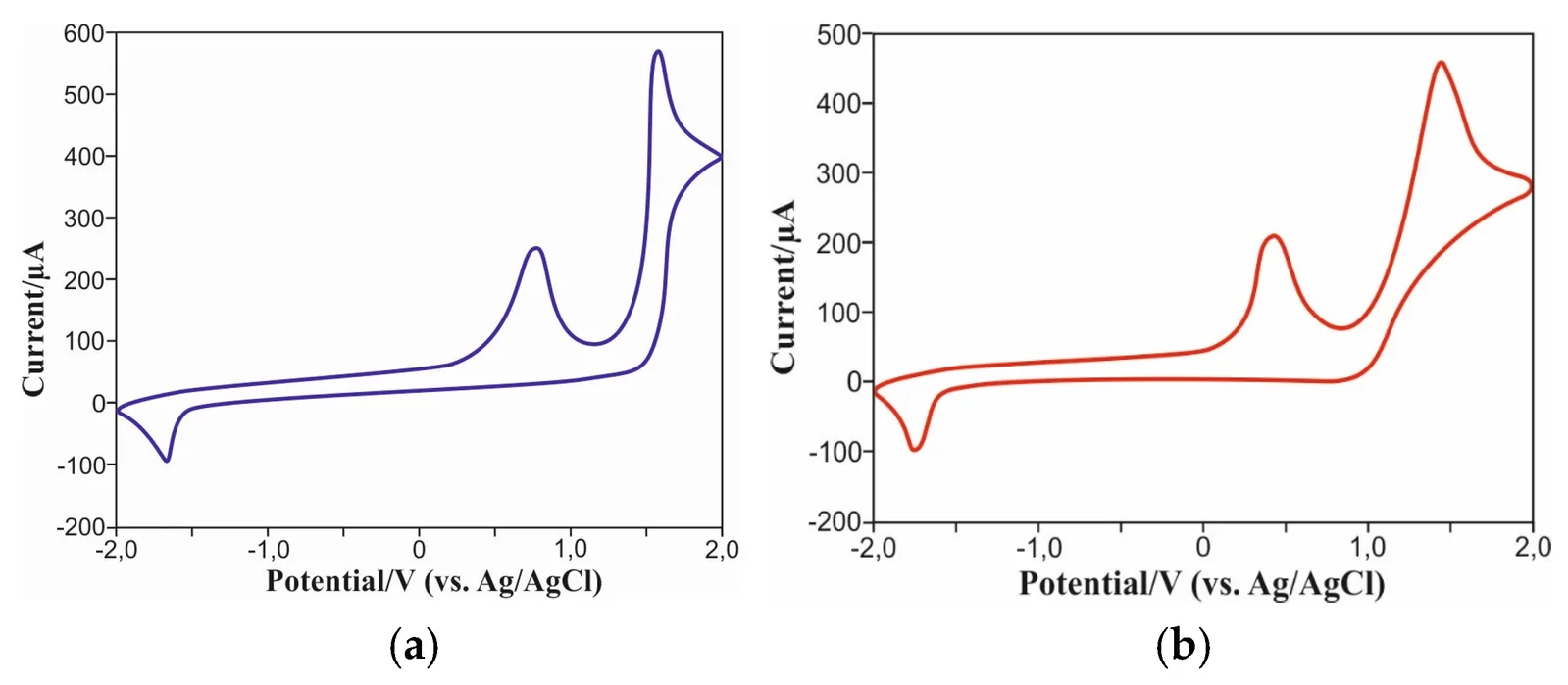
Cyclic voltammogram of 6 (**a**) and 10 (**b**).

To determine the energy levels of the frontal MO of the carbazolylazulenes **6** and **10**, a CV (cyclic voltammetry) analysis of their redox behavior was carried out (Figure 6, Supplementary Materials).

Analysis of CV curves revealed irreversible double oxidation peaks of the compound **6** at potentials of 0.77 V and 1.58 V. In addition, an irreversible single reduction peak was recorded at a potential of −1.67 V. The compound **10** also exhibited similar behavior, with irreversible oxidation at potentials of 0.43 V and 1.44 V, as well as an irreversible reduction wave observed at −1.75 V. Determining the initial oxidation (0.1 V for Eox^onset^) and reduction (−1.57 V for Ered^onset^) potentials, we calculated the energy values of the frontal orbitals for the compound **6**: −5.10 eV for HOMO and −2.83 eV for LUMO. Taking into account the initial oxidation (0.08 V for Eox^onset^) and reduction (–1.50 V for Ered^onset^) potentials, the HOMO and LUMO energy levels for molecule **10** were determined to be −5.12 eV and −2.90 eV, respectively. The calculation of these values was performed on the basis of the formula set forth in [37].

Thus, experimentally determined values of HOMO energy levels for the compounds **6** and **10** demonstrate good consistency with the results obtained using the DFT method. The difference between theoretically calculated and experimentally measured values of LUMO energy levels is insignificant, being approximately 0.39 V and 0.35 V for the compounds **6** and **10**, respectively.

## 3. Materials and Methods

**1,2,3-Tribromoazulene 4**. To a solution of monobromazulene **3** (207 mg, 1.00 mmol) in dichloromethane (5 mL) was slowly poured a solution of NBS (389 mg, 2.2 mmol) in dichloromethane (25 mL) at −20 °C under argon and stirred for 1.5 h. Then the mixture was treated with NaHCO_3_ solution, the organic phase was washed with brine and dried with Na_2_SO_4_. The resulting solution was filtered through alumina. After concentration on a rotary evaporator and recrystallization from dichloromethane, 337 mg (92%) of a dark green solid was obtained. m.p. 132–133 °C. IR spectrum (ν, cm^−1^): 2920, 2851, 1581, 1462, 1385, 1269, 725. ^1^H NMR: δ 7.94 (d, J = 11.4 Hz, 2H^4,8^_Az_), 7.62–7.59 (m, 2H^5,7^_Az_), 7.33 (t, J = 10.2 Hz, 1H^6^_Az_). ^13^C NMR: δ 139.6, 137.5, 136.3, 135.5, 134.3, 134.2, 128.9, 125.6, 107.1. HRMS (ESI): calcd. for C_10_H_5_^79^Br_3_ [M]^+^: 361,7898, found: 361,7891.

**1,2,3-Tris(carbazolyl)azulene 6**. Tribromoazulene **4** (336 mg, 1.00 mmol) and carbazole **5** (554 mg, 3.6 mmol) were mixed in dry toluene (35 mL) under argon. Palladium (II) acetate (12 mg, 0.05 mmol), *t*-BuOK (941 mg, 16.8 mmol), and *t*-Bu_3_PHBF_4_ (3.0 mg, 0.01 mmol) were then added. The mixture was then refluxed for 24 h, filtered through Celite and dried with Na_2_SO_4_. The solvent was removed by rotary evaporation. The pure compound was obtained by purification on silica gel using a 1:9 solution of dichloromethane and hexane (CC with SiO_2_) as eluent. After purification, the compound was recrystallized from CH_2_CI_2_. 487 mg (78%) of a brown solid were obtained. Melting point: 142–143 °C. IR spectrum (ν, cm^−1^): 2920, 2850, 1583, 1462, 1380, 1269, 724. ^1^H NMR: δ 8.40 (d, *J* = 9.6 Hz, 2H^4,8^_Az_), 8.09–8.03 (m, 6H^1,8^_Cz_), 7.91–7.85 (m, 2H^5,7^_Az_), 7.62–7.53 (m, 6H^4,5^_Cz_,1H^6^_Az_), 7.40–7.33 (m, 12H^2,3,6,7^_Cz_). ^13^C NMR: δ 138.2, 134.8, 134.2, 131.7, 130.8, 126.6, 124.3, 123.6, 123.1, 122.7, 122.2, 118.2, 117.1, 114.9, 112.5. HRMS (ESI): calcd. for C_46_H_29_N_3_ [M]^+^: 623,0720, found: 623,0712.

**1,2,3,6-Tetrabromoazulene 9.** To a solution of dibromoazulene **8** (286 mg, 1.00 mmol) in dichloromethane (5 mL) was slowly poured a solution of NBS (389 mg, 2.2 mmol) in dichloromethane (25 mL) at −20 °C under argon. The mixture was then stirred for 2 h. A saturated aqueous solution of sodium hydrogen carbonate was then added, after which the organic layer was separated and washed with brine until neutral. It was then dried over sodium sulfate. The resulting solution was filtered through alumina. Concentration on a rotary evaporator and recrystallization from dichloromethane gave 399 mg (90%) of a dark green solid. Batch 177–178 °C. IR spectrum (ν, cm^−1^): 2920, 2850, 1580, 1465, 1385, 1269, 725. ^1^H NMR: δ 8.29 (d, *J* = 9.7 Hz, 2H^4,8^_Az_), 7.33 (d, *J* = 9.9 Hz, 2H^5,7^_Az_).^13^C NMR: δ 139.6, 136.3, 135.5, 134,2, 130,9, 129.0, 125.6, 104.7. HRMS (ESI): calcd. for C_10_H_4_^79^Br_4_ [M]^+^: 440,0570, found: 440,0563.

**1,2,3,6-Tetrakis(carbazolyl)azulene 10.** Tetrabromazulene **9** (444 mg, 1.00 mmol) and carbazole 5 (738 mg, 4.8 mmol) were mixed in dry toluene (45 mL). The catalyst palladium (II) acetate (12 mg, 0.05 mmol), *t*-BuOK (1.254 g, 22.4 mmol), and *t*-Bu_3_PHBF_4_ (3.0 mg, 0.01 mmol) were then added to the mixture under continuous argon purge at ambient conditions. The mixture was then refluxed for 24 h, filtered through Celite and dried over sodium sulfate. The solvent was removed by rotary evaporation. The pure compound was obtained by purification on silica gel using a 1:9 solution of DCM and hexane (CC with SiO_2_) as eluent. After purification, the compound was recrystallized from CH_2_Cl_2_. 590 mg (75%) of a dark brown solid was obtained. Melting point: 146–148 °C. IR spectrum (ν, cm^−1^): 2920, 2854, 1580, 1461, 1384, 1268, 725. ^1^H NMR: δ 8.40 (d, *J* = 11.6 Hz, 2H^4,8^_Az_), 8.22–8.20 (m, 8H^1,8^_Cz_), 7.63–7.57 (m, 2H^5,7^_Az_),7.37–7.31 (m, 8H^4,5^_Cz_), 6.92–6.90 (m, 16H^2,3,6,7^_Cz_). ^13^C NMR: δ 139.9, 135.4, 134.4, 126,7, 126,6, 124.5, 124.3, 122.09, 122.06, 122.05, 121.4, 121.3, 120.7, 120.7, 110.1, 109.2, 106.6. HRMS (ESI): calcd. for C_58_H_36_N_4_ [M]^+^: 788,0685, found: 788,0678.

## 4. Conclusions

The synthesis of tris- and tetrakis(carbazolyl)azulenes **6** and **10** in high yields and their subsequent careful analysis showed that these compounds possess special optical characteristics. The inclusion of carbazolyl groups at positions 1,2,3, and 6 of azulene leads to pronounced absorbing and emitting properties in the visible spectrum (400–600 nm), in particular to intense emission in the region in the blue region, which is not demonstrated by the compound **1**. DFT calculations support these results by showing changes in the electronic structure that make possible π → π * electronic transitions previously qualified as prohibited. These conjugated compounds have great potential for applications in organic optoelectronics and materials science.

## Figures and Tables

**Table 1:** Photophysical properties of compounds **6** and **10**.

Compound	Absorption ^a^		Photoluminescence (PL) ^b^					
	λabs (nm)	ɛ (M^−1^ cm^−1^)	λ_PL_ (nm) ^c^	S_1_ (eV)	T_1_ (eV) ^d^	ΔE_ST_ (eV)	τ_p_/τ_d_ (ns/μs) ^e^	Φ_PL_^f^
**6**	295	88,888	444	3.06	2.79	0.27	7.4/4.6	0.12
	345	82,113						
	407	21,132						
**10**	267	71,057	490	2.87	2.63	0.24	8.7/19	0.25
	300	88,888						
	441	36,666						

^a^ Measured in dichloromethane at room temperature (C = 1 × 10^−4^ M). ^b^ λ_ex_ (excitation wavelength) 390 nm. ^c^ Measured in dichloromethane at room temperature. ^d^ Measured at 77 K dichloromethane. ^e^ Measured under N_2_ in dichloromethane at room temperature. ^f^ Absolute PLQY measured by integrating sphere under N_2_ in dichloromethane at room temperature

## Data Availability

The original contributions presented in this study are included in the article/Supplementary Material. Further inquiries can be directed to the corresponding author.

## Supplementary Materials

The following supporting information can be downloaded at: https://www.mdpi.com/article/10.3390/molecules30132797/s1, Figure S1: ^1^H NMR spectra of 1,2,3-tribromoazulene **4**.; Figure S2: ^13^C NMR spectra of 1,2,3-tribromoazulene **4**; Figure S3: HRMS spectra of 1,2,3-tribromoazulene **4**; Figure S4: ^1^H NMR spectra of 1,2,3-tris(carbazolyl)azulene **6**; Figure S5: ^13^C NMR spectra of 1,2,3-tris(carbazolyl)azulene 6; Figure S6: ^13^C NMR spectra of 1,2,3,6-tetrabromoazulene **9**; Figure S7: ^1^H NMR spectra of 1,2,3,6-tetrakis(carbazolyl)azulene **10**; Figure S8: ^13^C NMR spectra of 1,2,3,6-tetrakis(carbazolyl)azulene **10**; Figure S9: HRMS spectra of 1,2,3,6-tetrabromoazulene **9**; Figure S10: ^1^H NMR spectra of 1,2,3,6-tetrakis(carbazolyl)azulene **10**; Figure S11: ^13^C NMR spectra of 1,2,3,6-tetrakis(carbazolyl)azulene **10**; Figure S12: HRMS spectra of 1,2,3,6-tetrakis(carbazolyl)azulene **10**; Figure S13: Thermogravimetric (TGA) analysis of carbazolylazulene **6** and **10;** Table S1: Atomic coordinates of optimized geometry of **6**; Table S2: Atomic coordinates of optimized geometry of **10**.
