# Supplementary material for: Synthesis of Conjugated Tris- and Tetrakis (Carbazolyl) Azulenes with Intense Emission in the Visible Range
Source: Molecules. 2025 Jun 28;30(13):2797. doi: 10.3390/molecules30132797 (PMC12251248; doi:10.3390/molecules30132797)
Supplement: Supplementary file 1 [file molecules-30-02797-s001.zip › molecules-3697982-supplementary.pdf]

Supplementary Materials

Contents

1. Materials and Methods
2. Spectra of compounds
3. Thermogravimetric (TGA) analysis
4. Preparation of thin films and their absorption spectra
5. Density Functional Theory (DFT) Calculations
6. Cyclic voltammetry studies

1. Materials and Methods

The ^{1}H NMR and ^{13}C NMR spectra were recorded on a JNM-ECA 500 spectrometer (Jeol, Tokyo, Japan) (500 MHz and 126 MHz in $CDCl_3$, internal TMS standard). IR spectra were recorded on an Avatar-360 IR spectrometer (Thermo Nicolet, Waltham, MA, USA). High resolution mass spectra (HRMS) were obtained on a Thermo Electron Corporation DFS (Thermo Fisher Scientific Inc., Waltham, MA, USA) mass spectrometer. UV-visible spectra were measured on a Shimadzu UV-1800 spectrophotometer (Shimadzu, Kyoto, Japan). Photoluminescence spectra were measured on a FLS 1000 (Edinburgh Instruments, Edinburgh, UK) fluorescence spectrophotometer. CV was analyzed on a PalmSens instrument (PalmSens, Enschede, The Netherlands). Thermogravimetric analysis (TGA) was performed on a TGA instrument Q500 (TA Instruments, Newark, DE, USA). Melting points were obtained using a Buchi M-560 (Buchi, Luzern, Switzerland).

Commercially available reagents and solvents azulene, carbazole, $(Bpin)_2$, $[Ir(cod)\ Cl]_2$, $Pd(OAc)_2$, 2,2′ -bpy, CuBr, N-bromosuccinimide (NBS), t-BuOK, $t\text{-}Bu_3PHBF_4$, DMF, toluene, dichloromethane and others were used as available.

2. Spectra of compounds

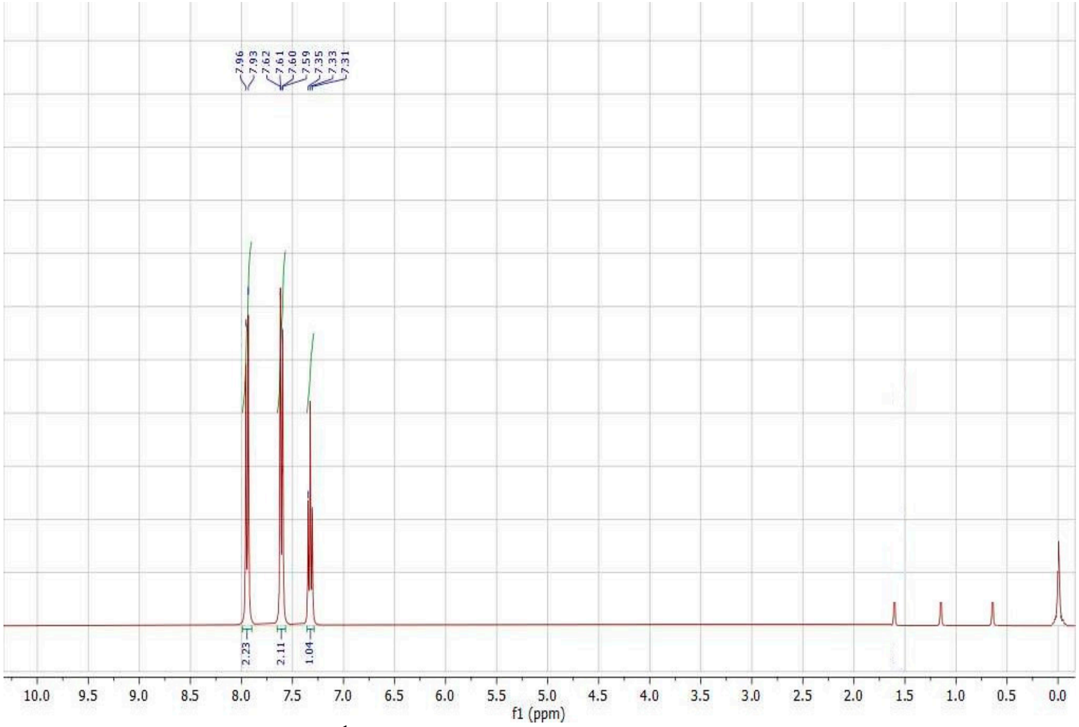

Figure S1. ^{1}H NMR spectra of 1,2,3-tribromoazulene **4.**

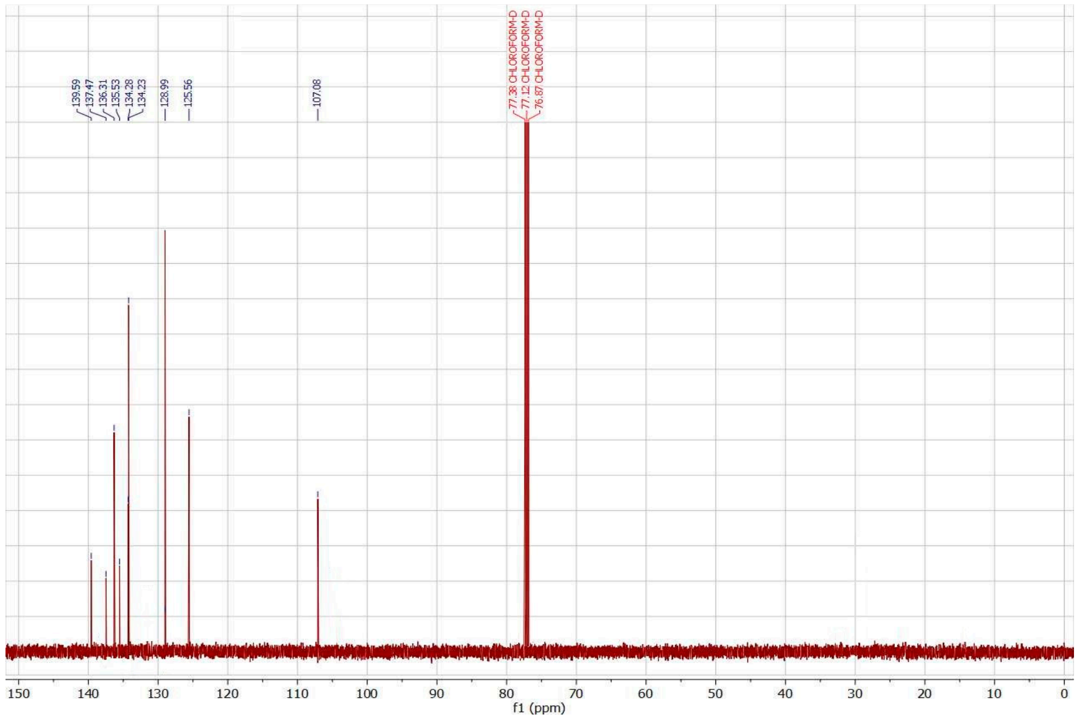

Figure S2. ^{13}C NMR spectra of 1,2,3-tribromoazulene **4**

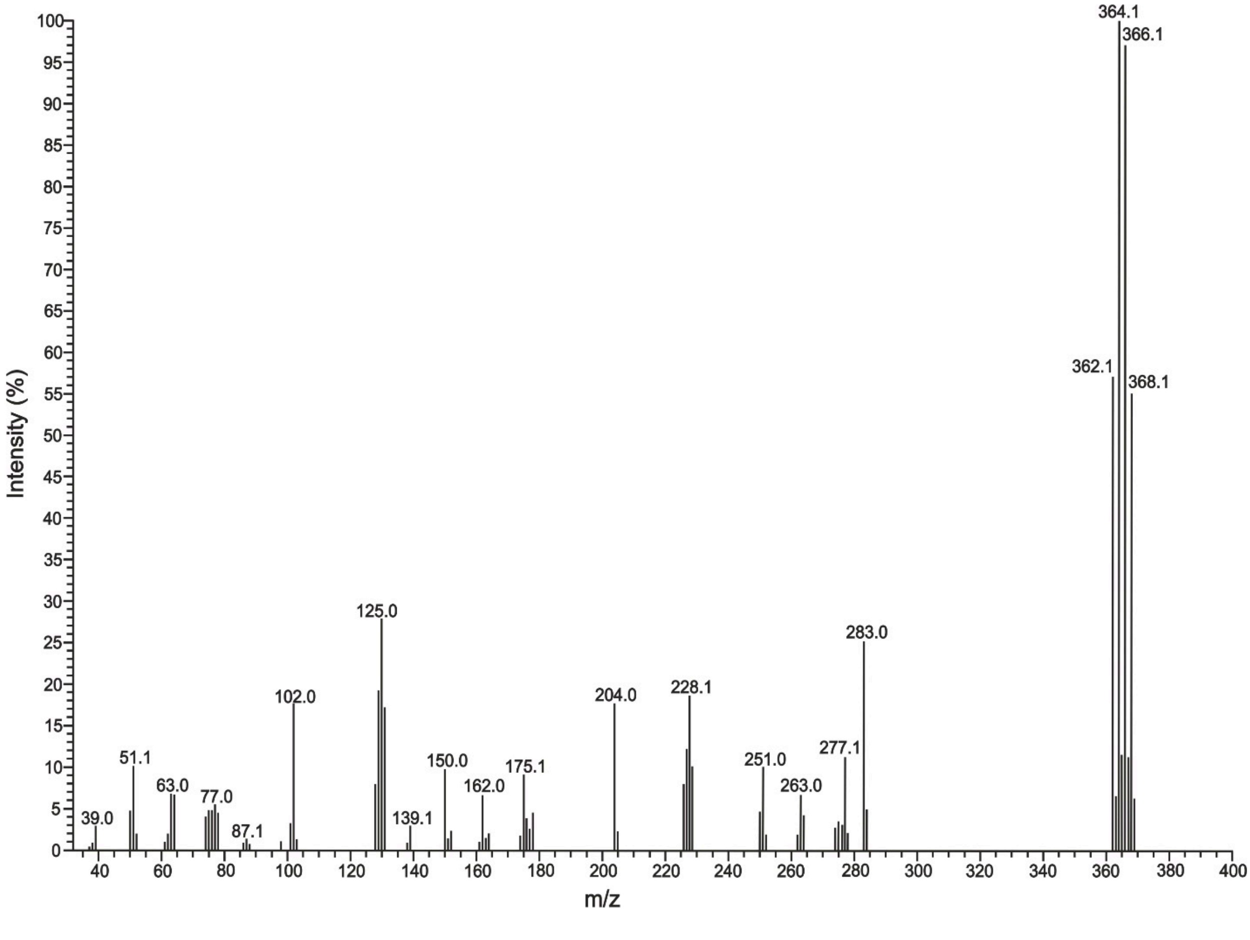

Figure S3. HRMS spectra of 1,2,3-tribromoazulene **4**.

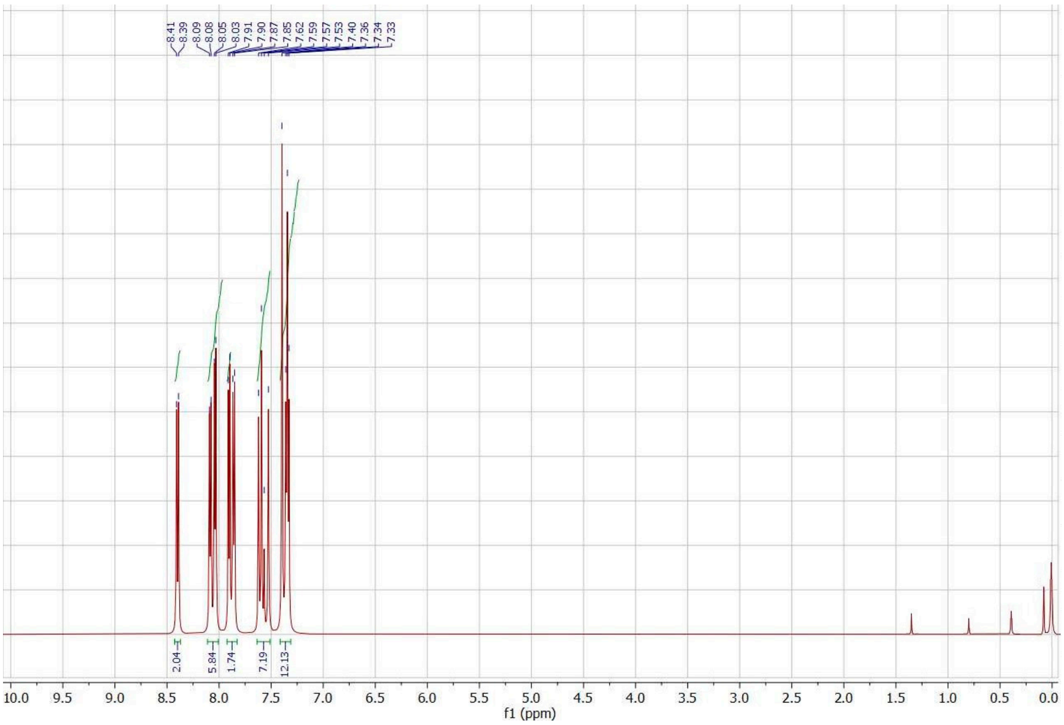

Figure S4. ^{1}H NMR spectra of 1,2,3-tris(carbazolyl)azulene **6**

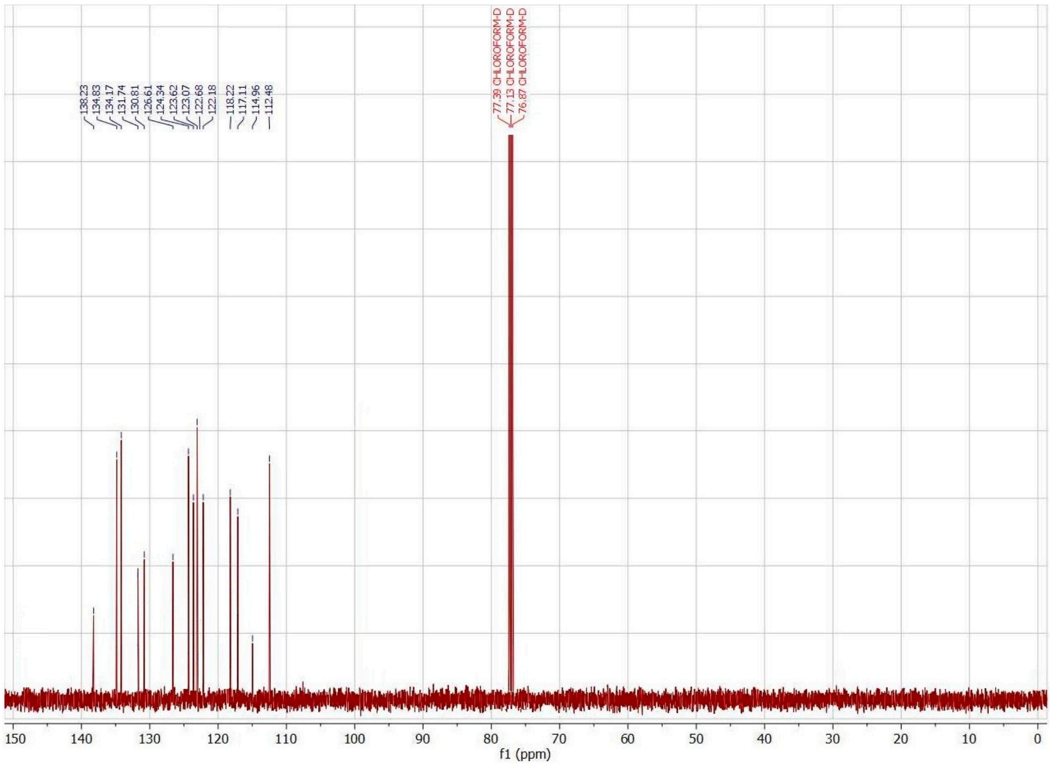

Figure S5. ^{13}C NMR spectra of 1,2,3-tris(carbazolyl)azulene **6**

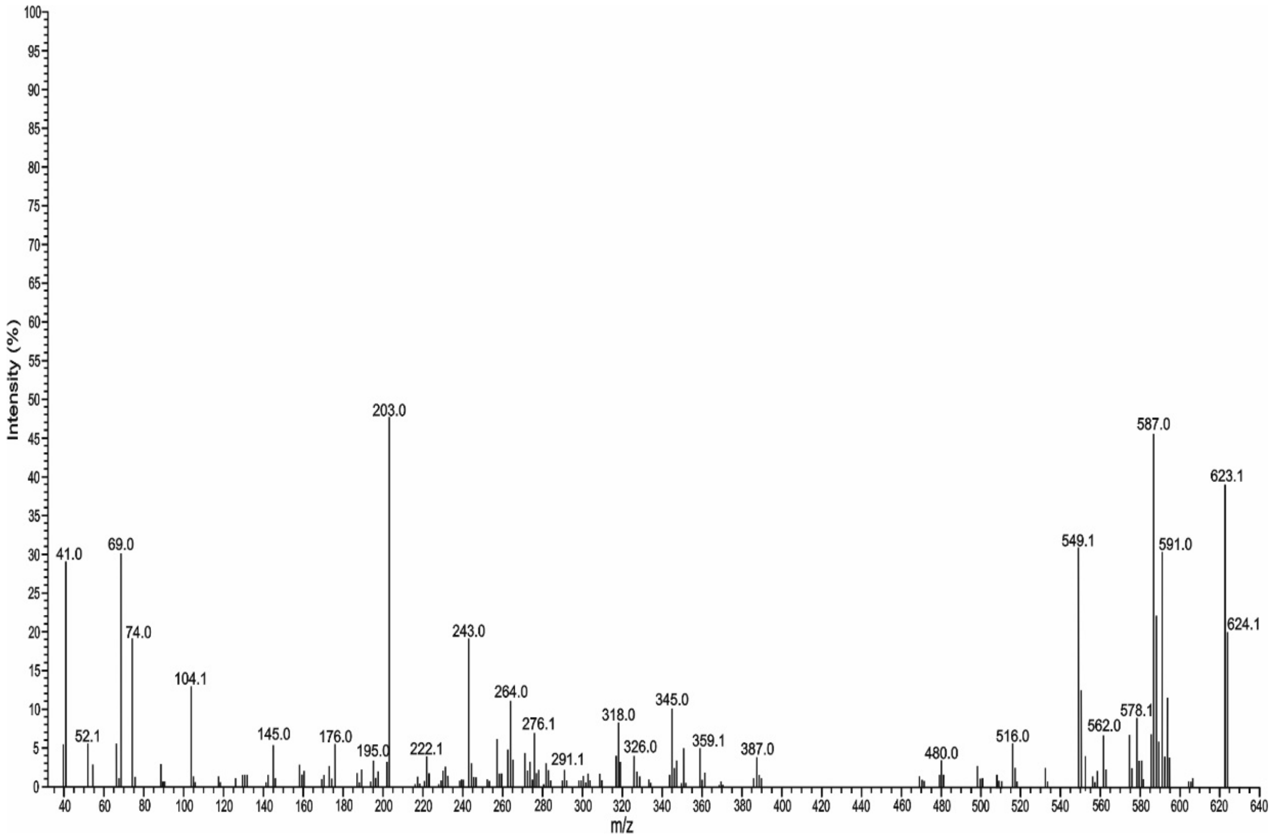

Figure S6. HRMS spectra of 1,2,3-tris(carbazolyl)azulene **6**.

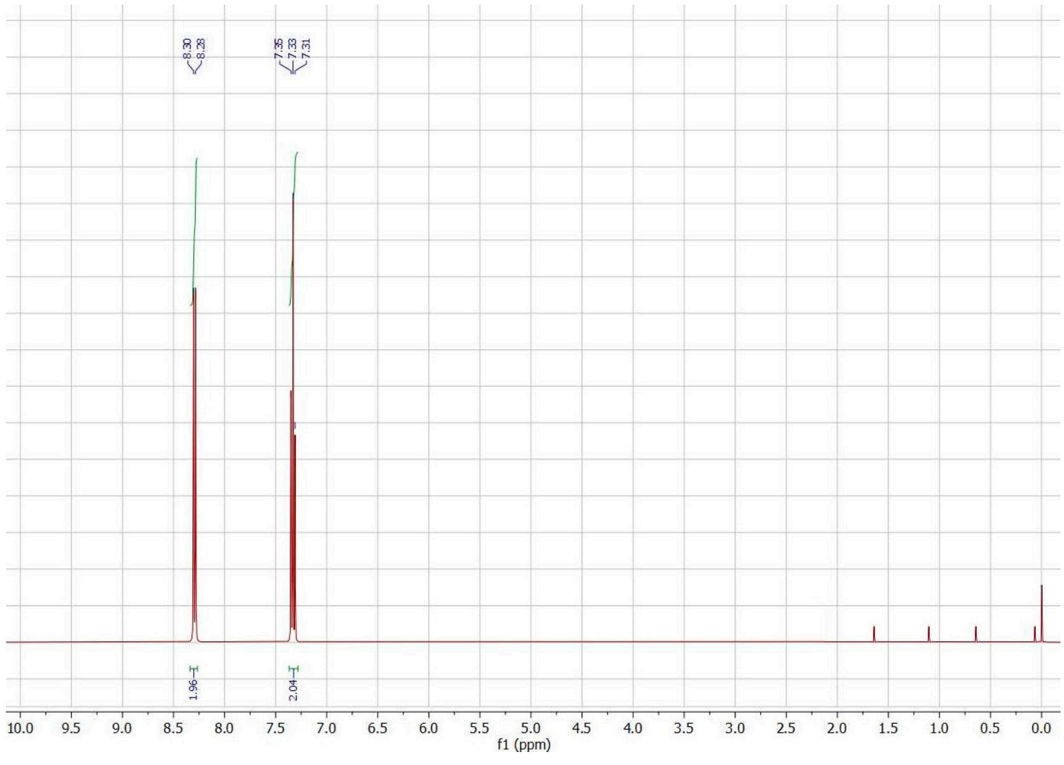

Figure S7. ^{1}H NMR spectra of 1,2,3,6-tetrabromoazulene **9**

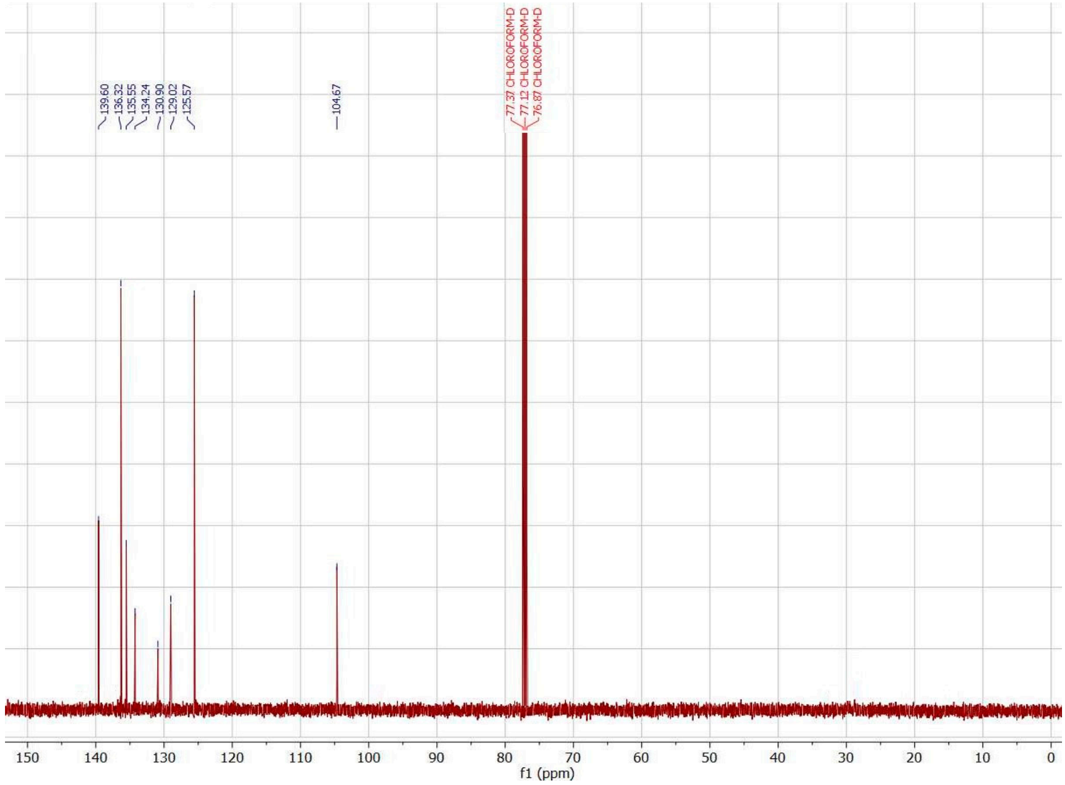

Figure S8. ^{13}C NMR spectra of 1,2,3,6-tetrabromoazulene **9**

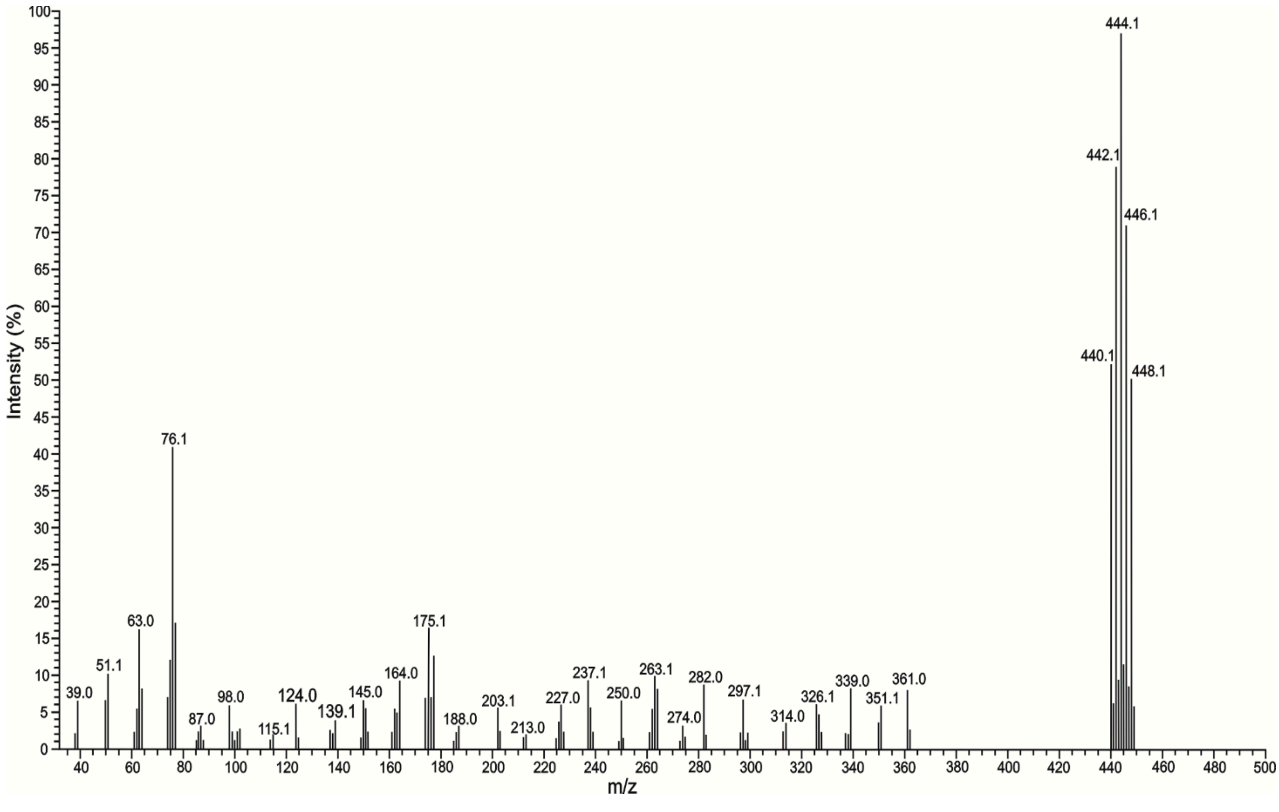

Figure S9. HRMS spectra of 1,2,3,6-tetrabromoazulene **9**

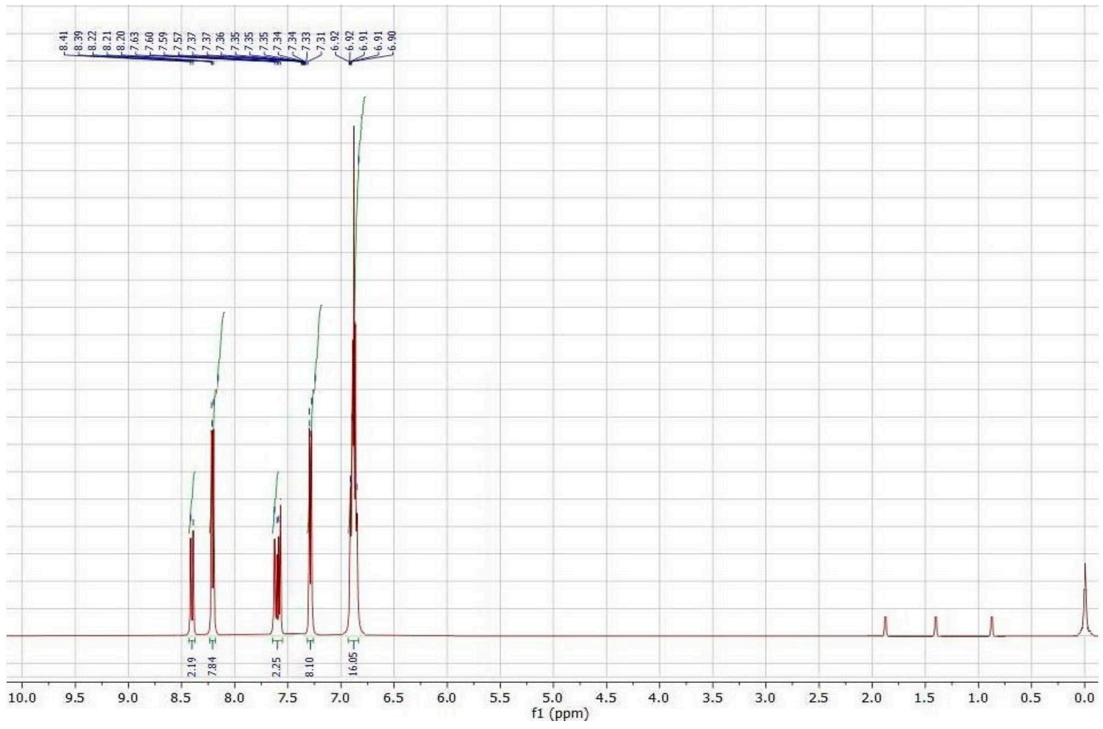

Figure S10. ^{1}H NMR spectra of 1,2,3,6-tetrakis(carbazolyl)azulene **10**

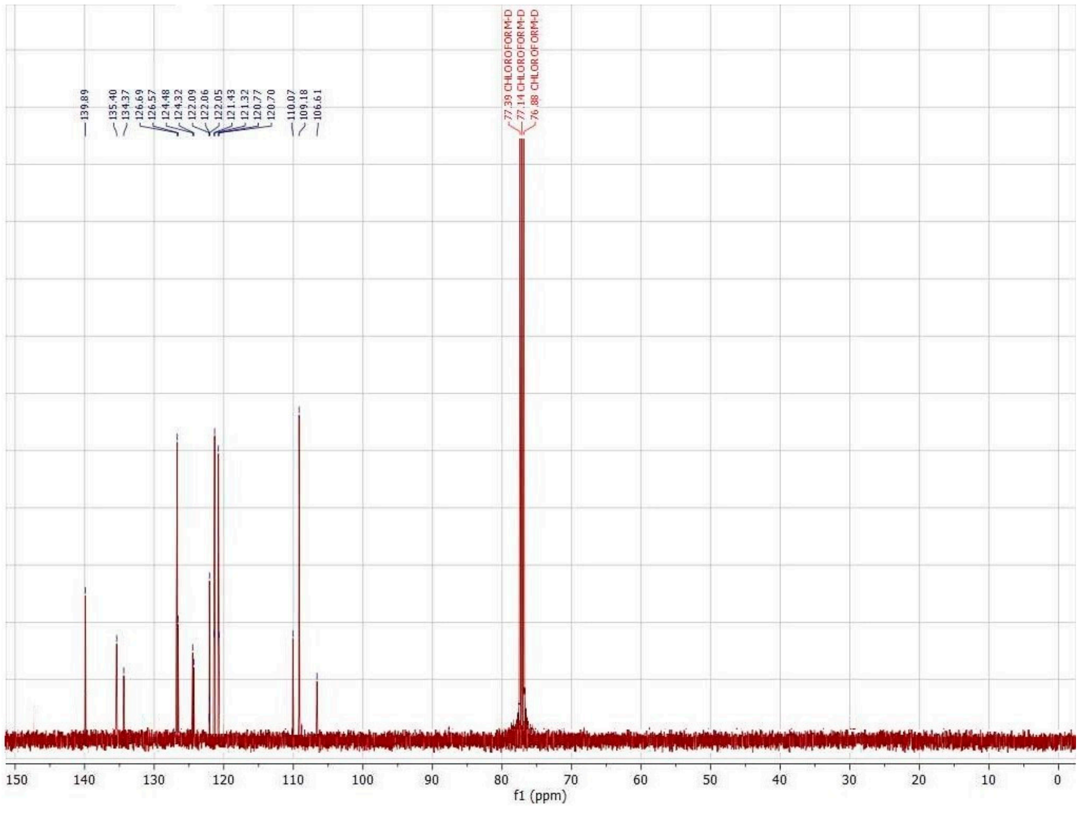

Figure S11. ^{13}C NMR spectra of 1,2,3,6-tetrakis(carbazolyl)azulene **10**

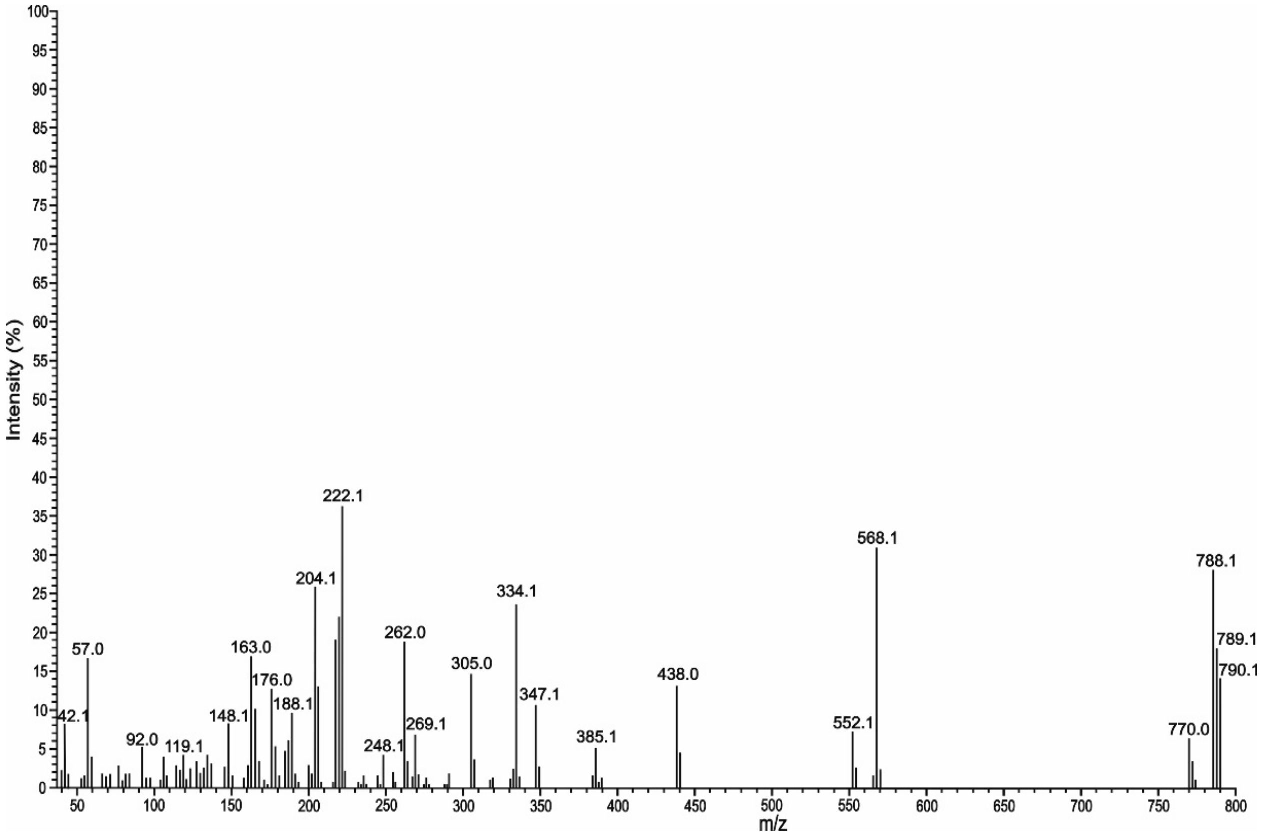

Figure S12. HRMS spectra of 1,2,3,6-tetrakis(carbazolyl)azulene **10**

3. Thermogravimetric (TGA) analysis

The thermal stability of compounds **6** and **10** was investigated by TGA analysis under nitrogen flow at a heating rate of 10°C per minute (range 20-500°C). The onset of degradation of the compounds (at a weight loss of 5%) was recorded at 321°C (for **10**) and 330 °C (for **6**), showing good thermal stability (figure S13).

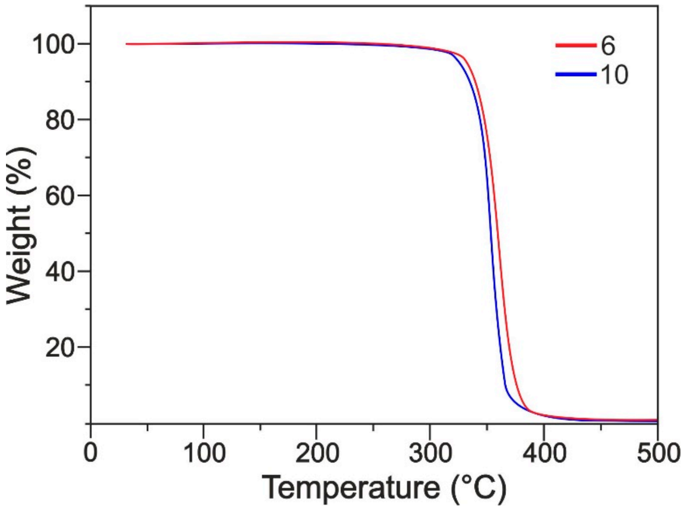

Figure S13. Thermogravimetric (TGA) analysis of carbazolylazulene **6** and **10**

4. Preparation of thin films and their absorption spectra

Thin films of трис - и тетра-carbazolylazulenes **6** and **10** were prepared by spin-coating a solution of each compound (10 mg/ml in chlorobenzene) at 1000 rpm for 60 seconds.

5. Density Functional Theory (DFT) Calculations

To characterize the geometry and orbitals of HOMO-LUMO трис- и тетра-carbazolylazulenes **6** and **10**, DFT calculations based on the level of B3LYP/6-31G * (d, p) Gaussian 16 were used. The geometry of the molecules was optimized in a singlet state using the keywords Opt and Freq, and the results were recorded in a .chk file to visualize the boundary orbitals. After the correct completion of the calculations, the .chk file was opened in GaussView 6.0 and the HOMO-LUMO orbitals were visualized from it using the built-in GaussView tool. Then we obtained the energies of the boundary orbitals in Hartree units and converted them to eV, multiplying by the conversion factor 27.2114

Table S1. Atomic coordinates of optimized geometry of **6**.

Center Number	Atomic Number	Atomic Type	Coordinates (Angstroms) X	Y	Z
1	6	0	-0.000085	5.574620	-0.000080
2	6	0	1.240497	4.977050	0.258214
3	6	0	1.570197	3.617551	0.283220
4	6	0	-1.240650	4.977005	-0.258348
5	6	0	0.737728	2.512129	0.099105
6	6	0	-1.570313	3.617495	-0.283293
7	6	0	-0.737814	2.512105	-0.099127
8	1	0	-0.000100	6.662782	-0.000104
9	1	0	2.063866	5.661508	0.444585
10	1	0	2.616871	3.385787	0.464733
11	1	0	-2.064038	5.661432	-0.444749
12	1	0	-2.616981	3.385693	-0.464797
13	6	0	1.150431	1.157635	0.126893
14	6	0	-0.000014	0.328113	0.000040
15	6	0	-1.150482	1.157599	-0.126861
16	6	0	-3.558024	0.951167	0.570004
17	6	0	-2.969457	-0.001195	-1.424657
18	6	0	-3.570933	1.614225	1.801895
19	6	0	-4.734324	0.367736	0.022708
20	6	0	-2.290965	-0.438017	-2.565303
21	6	0	-4.360113	-0.236097	-1.244392
22	6	0	-4.787377	1.691620	2.487148
23	1	0	-2.670367	2.058149	2.211078
24	6	0	-5.941281	0.460422	0.728577
25	6	0	-3.025371	-1.130719	-3.531348
26	1	0	-1.230937	-0.257151	-2.696258
27	6	0	-5.075248	-0.932650	-2.228214
28	6	0	-5.962349	1.122893	1.958227
29	1	0	-4.824204	2.200919	3.445006
30	1	0	-6.848420	0.019878	0.326658
31	6	0	-4.402719	-1.378091	-3.367660
32	1	0	-2.520979	-1.486981	-4.423760
33	1	0	-6.137383	-1.121881	-2.106967
34	1	0	-6.891320	1.200445	2.513493
35	1	0	-4.943495	-1.921123	-4.135799
36	6	0	-0.720770	-1.906709	0.892140

37	6	0	0.720846	-1.906668	-0.892051
38	6	0	-1.533751	-1.557794	1.973060
39	6	0	-0.452806	-3.263655	0.567862
40	6	0	1.533830	-1.557703	-1.972953
41	6	0	0.452931	-3.263629	-0.567796
42	6	0	-2.104246	-2.593496	2.719350
43	1	0	-1.732167	-0.523787	2.224341
44	6	0	-1.033834	-4.284205	1.330972
45	6	0	2.104371	-2.593371	-2.719255
46	1	0	1.732212	-0.523683	-2.224211
47	6	0	1.034004	-4.284145	-1.330918
48	6	0	-1.861629	-3.943379	2.403034
49	1	0	-2.748761	-2.346754	3.556775
50	1	0	-0.838433	-5.325792	1.095946
51	6	0	1.861798	-3.943268	-2.402967
52	1	0	2.748887	-2.346591	-3.556668
53	1	0	0.838643	-5.325743	-1.095911
54	1	0	-2.320019	-4.724407	3.000691
55	1	0	2.320223	-4.724269	-3.000633
56	6	0	2.969477	-0.001033	1.424704
57	6	0	3.557945	0.951176	-0.570060
58	6	0	2.291041	-0.437776	2.565413
59	6	0	4.360131	-0.235924	1.244401
60	6	0	3.570788	1.614120	-1.802013
61	6	0	4.734278	0.367812	-0.022765
62	6	0	3.025500	-1.130387	3.531483
63	1	0	1.231016	-0.256917	2.696400
64	6	0	5.075320	-0.932385	2.228249
65	6	0	4.787201	1.691476	-2.487326
66	1	0	2.670196	2.057989	-2.211199
67	6	0	5.941203	0.460458	-0.728694
68	6	0	4.402846	-1.377748	3.367758
69	1	0	2.521151	-1.486586	4.423945
70	1	0	6.137452	-1.121608	2.106972
71	6	0	5.962206	1.122820	-1.958403
72	1	0	4.823977	2.200688	-3.445231
73	1	0	6.848367	0.019963	-0.326776
74	1	0	4.943664	-1.920709	4.135917
75	1	0	6.891152	1.200338	-2.513717
76	7	0	0.000015	-1.076036	0.000044
77	7	0	2.481945	0.723433	0.314698
78	7	0	-2.481981	0.723362	-0.314686

Method: DFT B3LYP 6-31G*
Key word: opt freq
E(RB3LYP)= -1934.188213 Hartree

Table S2. Atomic coordinates of optimized geometry of **10**.

Center Number	Atomic Number	Atomic Type	Coordinates (Angstroms) X	Y	Z
1	6	0	3.748018	-0.000051	-0.000125
2	6	0	3.133440	-1.256669	0.136888
3	6	0	1.780278	-1.578247	0.148148
4	6	0	3.133403	1.256717	-0.137218
5	6	0	0.673988	-0.741053	0.038859
6	6	0	1.780392	1.578335	-0.148475
7	6	0	0.673941	0.741141	-0.039045
8	1	0	3.818267	-2.094694	0.200879

9	1	0	1.551877	-2.637719	0.222235
10	1	0	3.818332	2.094655	-0.201260
11	1	0	1.551952	2.637795	-0.222671
12	6	0	-0.676787	-1.156868	0.037166
13	6	0	-1.508236	-0.000048	0.000081
14	6	0	-0.676657	1.156931	-0.037155
15	6	0	-0.843984	3.499534	0.848271
16	6	0	-1.854958	3.073282	-1.160672
17	6	0	-0.159755	3.414661	2.061519
18	6	0	-1.425443	4.719335	0.406958
19	6	0	-2.337994	2.493679	-2.332299
20	6	0	-2.065208	4.448414	-0.871123
21	6	0	-0.057057	4.571069	2.835532
22	1	0	0.283918	2.482012	2.385859
23	6	0	-1.307582	5.865427	1.198182
24	6	0	-3.043809	3.306648	-3.218042
25	1	0	-2.185081	1.443732	-2.541726
26	6	0	-2.774467	5.245619	-1.774231
27	6	0	-0.622480	5.785534	2.410029
28	1	0	0.470847	4.529980	3.781195
29	1	0	-1.747346	6.801826	0.874772
30	6	0	-3.261174	4.668753	-2.945443
31	1	0	-3.436145	2.875871	-4.131655
32	1	0	-2.942101	6.295878	-1.565012
33	1	0	-0.524461	6.666649	3.032738
34	1	0	-3.815575	5.272505	-3.653885
35	6	0	-3.737895	0.691674	0.916804
36	6	0	-3.737944	-0.691633	-0.916592
37	6	0	-3.398510	1.468125	2.022885
38	6	0	-5.093587	0.432688	0.584664
39	6	0	-3.398608	-1.468202	-2.022605
40	6	0	-5.093618	-0.432565	-0.584443
41	6	0	-4.434731	2.004679	2.787990
42	1	0	-2.367946	1.672269	2.275292
43	6	0	-6.115937	0.977325	1.364872
44	6	0	-4.434868	-2.004735	-2.787674
45	1	0	-2.368055	-1.672466	-2.274973
46	6	0	-6.116004	-0.977181	-1.364615
47	6	0	-5.780199	1.765501	2.464630
48	1	0	-4.190912	2.622270	3.644241
49	1	0	-7.153911	0.783866	1.120267
50	6	0	-5.780320	-1.765437	-2.464334
51	1	0	-4.191094	-2.622410	-3.643877
52	1	0	-7.153966	-0.783655	-1.120008
53	1	0	-6.562163	2.197051	3.077867
54	1	0	-6.562313	-2.196971	-3.077544
55	6	0	-1.854553	-3.073445	1.160875
56	6	0	-0.844267	-3.499368	-0.848484
57	6	0	-2.337258	-2.494007	2.332719
58	6	0	-2.064767	-4.448568	0.871249
59	6	0	-0.160530	-3.414273	-2.061995
60	6	0	-1.425436	-4.719281	-0.407094
61	6	0	-3.042694	-3.307140	3.218616
62	1	0	-2.184389	-1.444063	2.542195
63	6	0	-2.773644	-5.245935	1.774514
64	6	0	-0.058006	-4.570580	-2.836182
65	1	0	0.282912	-2.481536	-2.386398
66	6	0	-1.307755	-5.865268	-1.198499
67	6	0	-3.260012	-4.669238	2.945950
68	1	0	-3.434770	-2.876496	4.132404
69	1	0	-2.941252	-6.296187	1.565239
70	6	0	-0.623129	-5.785157	-2.410600
71	1	0	0.469517	-4.529321	-3.782050

72	1	0	-1.747301	-6.801750	-0.875036
73	1	0	-3.814114	-5.273119	3.654516
74	1	0	-0.525258	-6.666187	-3.033452
75	7	0	-2.906827	0.000027	0.000076
76	7	0	-1.104783	-2.497481	0.111761
77	7	0	-1.104788	2.497499	-0.111745
78	6	0	6.002926	-0.607992	0.972367
79	6	0	6.003005	0.607906	-0.972559
80	6	0	5.664707	-1.283126	2.145953
81	6	0	7.360771	-0.384066	0.617496
82	6	0	5.664910	1.282987	-2.146213
83	6	0	7.360827	0.384105	-0.617500
84	6	0	6.700408	-1.759139	2.949717
85	1	0	4.631187	-1.428365	2.430066
86	6	0	8.382736	-0.868021	1.438128
87	6	0	6.700688	1.759076	-2.949839
88	1	0	4.631425	1.428112	-2.430512
89	6	0	8.382866	0.868097	-1.438014
90	6	0	8.046600	-1.560424	2.599949
91	1	0	6.458303	-2.288547	3.863647
92	1	0	9.421032	-0.701118	1.176125
93	6	0	8.046845	1.560469	-2.599888
94	1	0	6.458664	2.288459	-3.863804
95	1	0	9.421137	0.701239	-1.175881
96	1	0	8.828014	-1.943371	3.245031
97	1	0	8.828320	1.943482	-3.244855
98	7	0	5.175991	0.000005	-0.000133

Method: DFT B3LYP 6-31G*
Key word: opt freq
E(RB3LYP)= -2450.333289 Hartree

6. Cyclic voltammetry studies

Electrochemical studies were performed in an acetonitrile solution containing tetrabutylammonium hexafluorophosphate (nBu_4NPF_6) under an argon atmosphere. Ag/AgCl electrode, glassy carbon electrodes and platinum electrodes were used as reference electrodes, working electrodes and counter electrodes, respectively. 5 μl of a 0.8 mg/mL dichloromethane solution of carbazolylazulenes **6** (or **10**) was pipetted and dropped onto a glass-carbon electrode which was allowed to evaporate under the pressure of a washing flask at room temperature (the circular opening of the electrode had a diameter of 4 mm).
